# Next-generation sequencing yields a complete mitochondrial genome of the Asian Glass Lizard (*Dopasia gracillis*) from the Yungui Plateau in Southwest China

**DOI:** 10.1080/23802359.2020.1720537

**Published:** 2020-02-03

**Authors:** Bo Cai, Xianguang Guo, Jianping Jiang

**Affiliations:** a Chengdu Institute of Biology, Chinese Academy of Sciences, Chengdu, China;; b Key Laboratory of Bio-resources and Eco-environment of Ministry of Education, College of Life Sciences, Sichuan University, Chengdu, China;; c Chengdu Institute of Biology, University of Chinese Academy of Sciences, Beijing, China

**Keywords:** Anguidae, Asian Glass Lizard, mitochondrial genome, next-generation sequencing, phylogenetic tree

## Abstract

The Asian Glass Lizard, *Dopasia gracillis*, has wide distribution in North India, Nepal, South China, and Indochina. In this study, a complete mitochondrial genome of *D. gracillis* from the Yungui Plateau in Southwest China was determined by next-generation sequencing. Similar to the typical mtDNA of vertebrates, the mitogenome was 17,133 bp in length and comprised the standard set of 13 protein-coding genes (PCGs), 22 tRNA genes, 2 rRNA genes, and 1 control region. The concatenated PCGs were used to conduct Bayesian phylogenetic analyses together with mitogenome data of Anguidae and related taxa in GenBank. The resulting phylogenetic tree confirmed the monophyly of Anguidae and Aguinae as well as *D. gracillis*, respectively. The mitogenome reported here will contribute to the examination of phylogeographic structure for *D. gracillis* and understanding of mitochondrial DNA evolution in Anguidae.

The Asian Glass Lizard, *Dopasia (Ophisaurus) gracilis* is widespread in North India, Nepal, South China, and Indochina (Nguyen et al. 2011; Uetz et al. 2019). Despite its wide distribution, this lizard is considered to be a monotypic species without subspecies differentiation (Uetz et al. 2019). To date, little has been known about its population structure and genetic relationships with other congeneric species (Lavin and Girman 2019).

Recently, one mitochondrial genome (mitogenome) sequence of *D. gracilis* has been sequenced using Sanger sequencing (Yan 2015). In this study, we determined a complete mitogenome of this species using next-generation sequencing through the Illumina HiSeq 2000 platform. The specimen (Field number ML01) was collected from Mile city, Yunnan province, China on August 28, 2019. Its liver tissue was fixed with 95% ethanol and stored at –20 °C in the herpetological collection, Chengdu Institute of Biology, Chinese Academy of Sciences. A small amount of liver tissue was shipped to Tsingke (Chengdu, China) for genomic extraction and 150-base-pair paired-end library construction; sequencing was performed on an Illumina HiSeq 2000 instrument. *De novo* assembly of clean reads was performed using SPAdes v3.11.0 (Bankevich et al. 2012). The mitogenome of *D. gracilis* (GenBank accession number KJ941042) was further used as a reference to assemble the newly sequenced sample. Genes were annotated with the MITOS web server (Bernt et al. 2013).

The mitogenome of *D. gracilis* is 17,133 bp in length, comprising 13 protein-coding genes (PCGs), 22 tRNA genes, 2 rRNA genes, and 1 control region (CR or D-loop). The gene content, arrangement, and composition exhibited a typical vertebrate mitogenome feature. The majority of the genes in the mtDNA of *D. gracilis* was distributed on H-strand, except for the *ND6* and eight tRNA genes (*tRNA-Gln*, *Ala*, *Asn*, *Cys*, *Tyr*, *Ser^UCN^*, *Glu*, and *Pro*). In 13 PCGs, the shortest was ATP8 gene (168 bp) and the longest was *ND5* gene (1824 bp). Twelve of 13 PCGs were initiated with the typical ATG codon, except for *COX1* with GTG. Meanwhile, most PCGs were terminated with the typical TAA/TAG/AGG codons, except for *COX2*, *COX3, ND3,* and *ND4* with the incomplete termination codon T. The 22 tRNA genes ranged in size from 61 bp in *tRNA-Ser^AGY^* to 73 bp in *tRNA-Leu^UUR^* and *tRNA-Asn*. The *12S rRNA*, *16S rRNA*, and *D-loop* were 948 bp, 1559 bp, and 1691 bp in length, respectively.

The concatenated PCGs of Anguidae available in GenBank and *Heloderma suspectum* (Helodermatidae) plus *Shinisaurus crocodilurus* (Shinisauridae) as outgroups were used to reconstruct the Bayesian phylogenetic tree for assessing mitochondrial sequence authenticity of *D. gracilis* and its phylogenetic placement. As shown in Figure 1, the monophyly of both Anguidae and Anguinae was recovered (Lavin and Girman 2019; and references therein). Two individuals of *D. gracilis* clustered together and formed sister taxon to all other sampled congeners. The mitogenome sequence will provide fundamental data for further investigating the phylogeographic structure of *D. gracilis* along with exploring mitochondrial DNA evolution in Anguidae.

**Figure 1.**
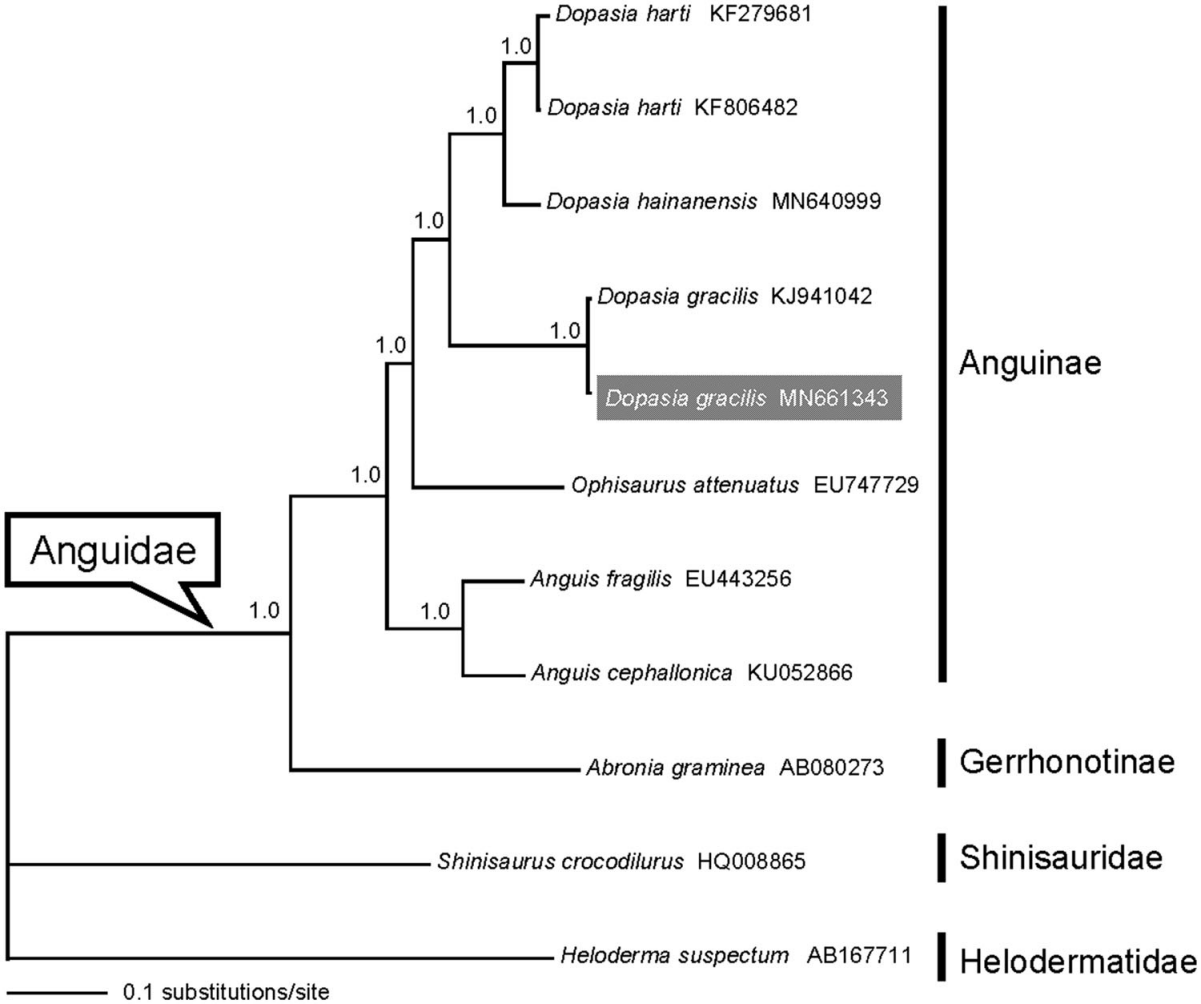
A majority-rule consensus tree inferred from Bayesian inference using MrBayes v.3.2.2 (Ronquist et al. 2012) under the GTR + G+I substitution model, based on the concatenated PCGs of nine lizards of Anguidae and two outgroup taxa representing Shinisauridae and Helodermatidae. The newly sequenced sample was highlighted in gray. DNA sequences were aligned in MEGA v.6.06 (Tamura et al. 2013). The PCGs were translated into amino acid sequences and were manually concatenated into a single nucleotide dataset (in total 11,463 bp). Node numbers show Bayesian posterior probabilities. Branch lengths represent means of the posterior distribution. GenBank accession numbers are given with species names.

## Nucleotide sequence accession number

The complete mitochondrial genome sequence of *Dopasia gracilis* has been assigned GenBank accession number MN661343.
